# Soluble and Cell-Associated Insulin Receptor Dysfunction Correlates with Severity of HAND in HIV-Infected Women

**DOI:** 10.1371/journal.pone.0037358

**Published:** 2012-05-22

**Authors:** Yamil Gerena, Richard L. Skolasky, Joyce M. Velez, Dianedis Toro-Nieves, Raul Mayo, Avindra Nath, Valerie Wojna

**Affiliations:** 1 NeuroAIDS Specialized Neuroscience Research Program, University of Puerto Rico, San Juan, Puerto Rico, United States of America; 2 School of Pharmacy, University of Puerto Rico, San Juan, Puerto Rico, United States of America; 3 Department of Orthopaedic Surgery, Johns Hopkins University, Baltimore, Maryland, United States of America; 4 Departments of Physical Medicine and Rehabilitation, University of Puerto Rico, San Juan, Puerto Rico, United States of America; 5 Section of Infections of the Nervous System, National Institute of Neurological Disorders and Stroke, National Institutes of Health, Bethesda, Maryland, United States of America; 6 Internal Medicine Neurology Division, University of Puerto Rico, San Juan, Puerto Rico, United States of America; South Texas Veterans Health Care System and University Health Science Center San Antonio, United States of America

## Abstract

**Background:**

Blood sugar metabolism abnormalities have been identified in HIV-infected individuals and associated with HIV-associated neurocognitive disorders (HAND). These abnormalities may occur as a result of chronic HIV infection, long-term use of combined antiretroviral treatment (CART), aging, genetic predisposition, or a combination of these factors, and may increase morbidity and mortality in this population.

**Objective:**

To determine if changes in soluble and cell-associated insulin receptor (IR) levels, IR substrate-1 (IRS-1) levels, and IRS-1 tyrosine phosphorylation are associated with the presence and severity of HAND in a cohort of HIV-seropositive women.

**Methods and Results:**

This is a retrospective cross-sectional study using patient database information and stored samples from 34 HIV-seropositive women and 10 controls without history of diabetes from the Hispanic-Latino Longitudinal Cohort of Women. Soluble IR subunits [sIR, ectodomain (α) and full-length or intact (αβ)] were assayed in plasma and CSF samples by ELISA. Membrane IR levels, IRS-1 levels, and IRS-1 tyrosine phosphorylation were analyzed in CSF white cell pellets (WCP) using flow cytometry.

HIV-seropositive women had significantly increased levels of intact or full-length sIR in plasma (p<0.001) and CSF (p<0.005) relative to controls. Stratified by HAND, increased levels of full-length sIR in plasma were associated with the presence (p<0.001) and severity (p<0.005) of HAND. A significant decrease in IRS-1 tyrosine-phosphorylation in the WCP was also associated with the presence (p<0.02) and severity (p<0.02) of HAND.

**Conclusions:**

This study provides evidence that IR secretion is increased in HIV-seropositive women, and increased IR secretion is associated with cognitive impairment in these women. Thus, IR dysfunction may have a role in the progression of HAND and could represent a biomarker for the presence and severity of HAND.

## Introduction

The prevalence of HIV-associated neurocognitive disorders (HAND) has increased due to the widespread use of combined antiretroviral treatment (CART) and resulting longer survival of HIV-infected patients [1], [2], [3]. In the United States, HAND's prevalence among HIV-infected individuals on CART is as high as 50%, and HAND continues to be a significant cause of morbidity in chronically infected patients [4], [5]. The majority of these patients suffer from the milder forms of HAND [5], [6], which can develop and progress despite adequate viremia control and good CART adherence [7], [8], [9], [10], [11]. In addition, blood sugar metabolism abnormalities, such as insulin resistance, have recently been identified in HIV-infected individuals and associated with HAND [12], [13], [14], [15]. Studies in the general population indicate that such abnormalities may precede the overt onset of diabetes by several years [16], [17], and are risk factors for cardiovascular and cerebrovascular outcomes [18].

Insulin receptor dysfunction may also contribute to cognitive impairment [19], [20], [21]. Insulin receptor and the type 1 insulin-like growth factor receptor have essential roles in energy homeostasis, neuronal growth and survival, and synaptic plasticity (Reviewed in [20], [22], [23], [24]). Insulin receptors are enriched in the hippocampus, and insulin has been shown to enhance learning and memory, and to promote hippocampal long-term potentiation [24]. Conversely, insulin resistance and diabetes have been associated with risk of cognitive impairment and dementia in the general population [19], [25], [26], [27], [28], [29] and in patients with HIV [12], [13].

While blood glucose abnormalities have been associated with the development of HAND, no studies to date have evaluated plasma sIR subunit expression in HAND. Exploring potential changes in sIR expression in HIV patients could provide insight into the pathogenesis of HAND.

Changes in plasma sIR expression might also serve as biomarkers for HAND. Since the advent of CART, the clinical spectrum of HAND has changed. Whereas several CSF inflammatory markers such as beta-2-microglobulin correlated with HIV-associated dementia (HAD) in the pre-CART era, in CART-treated individuals these markers show little correlation [3], [9], [10], [30], [31], [32], [33]. Hence, there is a critical need to identify alternative biomarkers for HAND, particularly its milder forms. Several studies are evaluating potential CSF and blood markers, including inflammatory markers, activated monocytes (CD16+), proviral HIV DNA, and *“omics”* profiles. Others are using neuroimaging methods such as magnetic resonance spectroscopy, functional MRI, diffusion tensor imaging, or resting cerebral blood flow [32], [34], [35], [36]. However, CSF sampling is invasive, and neuroimaging may be impractical or unfeasible in many settings [37]. Blood biomarkers would be ideal, as these can be most easily and non-invasively obtained. Plasma full-length sIR could serve as a blood biomarker for early stages of HAND. In addition, the effect of insulin resistance on cognition can be reversed by the administration of insulin [22]. Thus, identification of insulin resistance in HIV patients could open a treatment modality early in the course of HAND to lessen its severity and progression. We therefore performed a retrospective study to evaluate how soluble and cell-associated insulin receptor dysfunction might correlate with severity of HAND in HIV-infected women.

## Methods

### Objective

To determine if changes in soluble and cell-associated insulin receptor (IR) levels, IR substrate-1 (IRS-1) levels, and IRS-1 tyrosine phosphorylation are associated with the presence and severity of HAND in a cohort of HIV-seropositive women.

### Participants and study design

This is a retrospective cross sectional study using patient database information and the samples repository of the Hispanic-Latino Longitudinal Cohort of Women. This study is approved by the University of Puerto Rico Medical Sciences Campus (UPR MSC) Institutional Review Board and all participants signed a written informed consent. This cohort is part of the NeuroAIDS Specialized Neuroscience Research Program (SNRP) at the UPR, MSC. This is a unique cohort of Hispanic HIV-seropositive women characterized longitudinally with viral and immune profiles, neurological exams, and neuropsychological tests.

Thirty-four (34) HIV-seropositive women and 10 seronegative controls without history of diabetes were evaluated as described previously [38]. These participants fulfilled inclusion and exclusion criteria of the Hispanic-Latino Longitudinal Cohort of Women (described previously in [38]) with the inclusion criteria of (i) being ≥18 years old, (ii) having completed at least a 9^th^ grade education, and (iii) having a nadir CD4 cell count ≤500 cells/mm^3^ during the last year and/or a viral load >1,000 copies/mL while using HAART. Women with a history of neurodegenerative diseases or prior CNS infections (e.g., toxoplasmosis), psychiatric conditions, active infections, or head trauma were excluded. These criteria identified a group of women with HIV-1 infection who were at risk of developing HAND [39], [40]. The selection of participants did not rely on self-reported cognitive concerns. Participants underwent neurological and neuropsychological evaluations, blood sampling to determine viral immune profiles, and test for hepatitis C virus, and CSF sampling. The women were matched for age and education. Fasting blood sugar test (FBS) was normal in the 24 women who had the test performed for other reasons.

HIV-seronegative control samples were 5 plasma samples obtained from healthy donors and 5 CSF samples from Dr. Wojna's CSF repository at the UPR MSC. The CSF samples were obtained in the course of medical care such as surgical gynecological procedures where spinal anesthesia was performed, and in one case from a patient with Acute Demyelinating Encephalomyelitis (ADEM). The women who underwent spinal anesthesia were not receiving any medications prior to the spinal tap. Controls and HIV-seropositive women were matched for age.

### Description of Procedures undertaken

#### Ethics and Participant's evaluations

After giving their consent to take part in this IRB-approved research project, individual participants were required to provide demographic and medical history information along with having a macroneurological exam and specimens for laboratory analysis. The information included age, most likely mode of HIV transmission, nadir CD4 cell count, and actual antiretroviral treatment. Plasma and CSF viral load was determined via Ultrasensitive RNA Roche Amplicor at an AIDS Clinical Trials Group (ACTG)-Certified Laboratory.

#### Neurocognitive testing

Neurocognitive tests (described previously in [38], [41]) evaluated the following cognitive domains (neuropsychological [NP] tests/subtests): verbal memory (trial 5, delay recall, and recognition of a Spanish translation of the Rey Auditory Verbal Learning Test), frontal executive function (Stroop Color Word Test [word/color, Manual de Stroop. Copyright 1993, by TEA ediciones, S.A. Madrid (España)] and Trail Making B), psychomotor speed (Symbol Digit Modalities Test and visual and auditory reaction time non dominant hand), and motor speed (Trail Making A and Grooved Pegboard dominant and non dominant hand). Depressive symptoms were determined using the Beck Depression Index [42], [43]. The cognitive pre-morbid status between HIV-seropositive and HIV-seronegative women was determined using the Wechsler Adult Intelligence Test - Vocabulary subtest [44] and the Woodcock-Muniz Test - Reading subtest (W31) [45]. All tests were conducted in Spanish. We calculated z-scores for each NP tests/subtests using a reference control group of 34 HIV-seronegative healthy women that did not differ from the HIV-seropositive participants with regard to socio-economic status (mode <$5,000 annual income), education (p = 0.828), and pre-morbid status (reading and vocabulary performance, p = 0.649 and 0.190 respectively). A significant difference between reference control group and HIV-seropositive women was observed in age (p<0.001), where the mean age(SD) and range of the younger reference control group (n = 34) was of 34.2(6.9) years with a range of 22 to 49 years while the HIV+ women (n = 34) had 42.1(7.3) years with a range of 25 to 54 years, and in the Beck's Depression Index (BDI) where HIV-seropositive women presented with higher scores of depressive symptoms (p = 0.001). A z-score (using the mean and standard deviation of the reference control group) was calculated for each test, cognitive domain, and neuropsychological z-score (NPZ). The HIV-seropositive women group presented with a mean (SD) in the following socio-demographic characteristics age of 42.1(7.3) years, education of 12.7(2) years, and a pre-morbid status in reading of 27.9(2) and vocabulary of 47.6(11.7). Depressive symptoms as determined by Beck Depression Index of 10.9(9.8).

Cognitive impairment was defined according to the American Academy of Neurology (AAN) HIV criteria modified to identify asymptomatic cognitive impairment as described previously [38], [41], [46]. Using this scale we grouped the HIV-seropositive women into 11 with normal cognition, 8 with asymptomatic neurocognitive impairment, and 15 with symptomatic neurocognitive impairment (mild cognitive motor disorder [MCMD, n = 1] and HIV-associated dementia [HAD, n = 14]). No significant differences were observed in age, education, or socio-demographic factors between groups stratified by HAND (p = 0.570).

Stratifying by cognitive impairment no significant differences were observed in education levels (p = 0.515), reading and vocabulary scores (p = 0.987 and 0.633 respectively). Stratifying by cognitive performance, the symptomatic impairment group performed worse in most of the neuropsychological tests (p<0.005). (Table 1).

**Table 1:** HIV-seropositive women neuropsychological performance.

	Normal Cognition (n = 11)	Asymptomatic impairment (n = 8)	Symptomatic impairment (n = 15)	p-value^a^
bEducation, years	13.3(14.1) 12, 16	12.8(2.1) 9, 16	12.3(2.5) 9, 16	0.515
Reading scores	27.7(2.8) 21, 30	27.9(2.0) 23, 29	28.1(1.7) 25, 30	0.987
Vocabulary scores	49.9(7.2) 39, 62	46.6(10.5) 33, 62	46.4(15.1) 22, 79	0.633
cTrail Making A test	−0.1(1.1) −1.8, 1.3	−0.3(0.9) −1.5, 0.9	−0.6(0.8) −1.9, 1.2	0.431
Trail Making B test	0.6(0.6) −0.6, 1.4	−0.3(1.3) −3.1, 1.0	−0.7(1.4) −3.9, 1.1	0.015*
Symbol Digit Modality Test	0.0(1.0) −1.3, 1.6	−0.9(0.7) −2.1, 0.1	−1.3(0.6) −2.5, −0.2	0.005*
Grooved Pegboard dominant hand	0.2(0.5) −0.7, 0.8	−1.6(2.1) −5.8, 0.5	−0.3(0.6) −1.7, 0.6	0.039*
Grooved Pegboard non dominant hand	0.3(1.0) −1.9, 1.4	−2.9(3.1) −8.1, 0.8	−0.2(1.0) −2.2, 1.5	0.014*
Stroop color-word	0.2(1.0) −1.6, 1.5	−0.5(1.3) −2.6, 1.5	−1.5(1.5) −4.3, 0.9	0.013*
dRAVLT recognition	0.8(0.7) −0.9, 1.2	−0.1(0.8) −1.3, 1.2	−0.5(0.9) −2.2, 0.7	0.001*
RAVLT delay recall	0.4(0.9) −0.9, 1.8	−0.3(0.9) −1.6, 0.8	−0.7(1.0) −1.9, 1.1	0.028*
RAVLT trial 5	0.2(0.8) −1.0, 1.1	−0.7(0.8) −1.7, 0.4	−0.6(1.0) −2.4, 0.8	0.037*
Visual Reaction Time non dominant hand	0.3(0.8) −0.8, 1.5	0.9(0.9) −0.6, 1.8	−0.2(1.3) −3.0, 2.4	0.094
Auditory Reaction Time non dominant hand	0.3(0.4) −0.5, 0.9	0.3(0.4) −0.5, 0.8	−0.3(0.8) −2.8, 0.7	0.042*
Cube Copy	−0.3(1.1) −2.0, 0.9	−0.8(1.0) −2.0, 0.9	−0.6(0.6) −2.0, −0.1	0.617
fNPZ	0.3(0.4) −0.5, 1.0	−0.6(0.5) −1.4, 0.0	−0.7(0.4) −1.7, −0.1	<0.001*
gFrontal Executive Domain	0.4(0.6) −0.5, 1.4	−0.4(0.9) −1.5, 0.9	−1.1(1.2) −3.7, 1.0	0.002*
Psychomotor Speed Domain	0.2(0.5) −0.6, 0.9	0.1(0.5) −0.7, 0.7	0.7(0.7) −2.5, 0.1	0.006*
Verbal Memory Domain	0.5(0.7) −0.9, 1.4	−0.4(0.6) −1.5, 0.3	−0.6(0.8) −2.0, 0.8	0.005*
Motor Speed Domain	0.1(0.7) −0.9, 1.1	−1.6(1.4) −4.4, 0.1	−0.4(0.6) −1.4, 0.7	0.007*

a significant p value<0.05^*^,

b mean(SD), range,

c z-scores, mean(SD), range,

d RAVLT = Rey Auditory Verbal Learning Test,

f NPZ = average of the z-scores of all tests,

g cognitive domains.

#### HIV-seropositive women characteristics

When the HIV-seropositive women were grouped by cognitive performance, no significant differences were observed between groups with regard to CD4 cell count, plasma viral load, co-infection with Hepatitis C virus, body mass index (BMI), or toxicology (p>0.05). Significant differences were observed in nadir CD4 cell counts and CSF viral loads, with both being higher in the asymptomatic cognitively impaired group (p = 0.044 and 0.041 respectively; Table 2).

**Table 2:** HIV-seropositive women characteristics.

	Normal Cognition (n = 11)	Asymptomatic impairment (n = 8)	Symptomatic impairment (n = 15)	p-value^a^
Age, years	44.0 (39.0, 47.0)	42.0 (35.5, 44.5)	43.0 (41.0, 48.0)	0.570
bCD4 cells/mm^3^	397.0 (275.0, 459.0)	521.0 (346.5, 720.5)	423.0 (291.0, 613.0)	0.503
CD4 nadir cells/mm^3^	177.0 (14.0, 384.0)	439.0 (292.0, 513.4)	191.0 (111.0, 391.0)	0.044^*^
CSF HIV RNA (log)	1.7 (1.7, 1.7) Detected in 2/9	2.2 (1.7, 2.3) Detected in 4/6	1.7 (1.7, 1.7) Detected in 1/13	0.041^*^
Plasma HIV RNA (log)	1.7 (1.7, 3.3) Detected in 4/9	2.9 (2.1, 3.6) Detected in 5/6	1.7 (1.7, 2.2) Detected in 5/12	0.095
Hepatitis C virus	4/11	0/7	6/14	0.131
cBMI	26.5 (25.6, 27.4)	29.9 (22.7, 39.4)	25.6 (23.1, 28.2)	0.420
Toxicology	0/10	1/8; marijuana	3/15; 1 marijuana & 2 cocaine	0.874
ART	11/11 HAART	2/8 none, 2/8 ART, 4/8 HAART	1/15 ART, 14/15 HAART	0.077
Protease inhibitors	8/11 (72.7%)	5/8 (62.5%)	10/15 (66.6%)	0.893
dCPE	6.0 (4.0, 7.0) 3, 10	6.0 (4.0, 7.0) 2, 9	6.0 (4.0, 8.0) 1, 9	0.528

a significant p value<0.05^*^,

b median(interquartile range [25^th^ and 75^th^ percentile]),

c BMI = body mass index,

d CPE = CNS penetration Effect [4].

Most HIV-seropositive women were using CART, (94%, or 32 of the 34 women). There was no significant difference among groups with regard to their specific CART regimens. Using the CNS Penetration Effect (CPE) scale prepared by Dr. Scott Letendre [4], we observed no differences in CPE among groups (Table 2).

#### Blood sample preparation

Fresh blood samples were drawn from each patient and collected in ACD tubes. Plasma samples were obtained from blood double spun centrifugation at 355×g for 10 minutes at room temperature. A plasma aliquot of 700 µL was used for viral load determination, and aliquots of 500 µL were used to determine changes in soluble and cell-associated insulin receptor (IR) and IR substrate-1 (IRS-1).

#### CSF sample preparation

All spinal taps were performed by the same neurologist (VW) using an atraumatic Sprotte needle. CSF was centrifuged at 130×g for 10 minutes at 4°C, and cell pellets and supernatants were separated. Aliquots of supernatant were stored at −80°C and cell pellets were maintained in liquid nitrogen. CSF samples tested negative for VDRL.

#### Soluble insulin receptor full-length (sIR-αβ, intact) and insulin receptor-ectodomain (sIR-α) Assay

Plasma sIR ectodomain and full-length were assayed as described by The Soluble Insulin Receptor Study Group [47] using an anti-IR ectodomain antibody (1∶1000) (Abcam, Cambridge, MA) for 2 hours at 20°C, and a FITC-secondary antibody (Abcam, Cambridge, MA). Samples were analyzed in a Cytofluor 4000 (Applied Byosystems, CA) using 485/530 nm excitation/emission filters.

#### Membrane-bound insulin receptor (mIR) levels, IRS-1 levels and IRS-1 tyrosine phosphorylation

In a subgroup of the patients described above (23 stratified by HAND into 7 women with normal cognition, 7 with asymptomatic impairment, and 9 with symptomatic impairment), we analyzed mIR levels, IRS-1 levels, and IRS-1 tyrosine phosphorylation in CSF cell pellets (5 per HAND group). For determination of mIR levels, pellets (1×10^6^cells) were incubated with anti-IR-PE antibody (BD biosciences, CA) for 1 hour at 4°C. For detection of intracellular IRS-1 levels and phosphorylation, pellets (1×10^6^ cells) were permeabilized using the BD Cytofix\Cytoperm Kit (BD biosciences, CA), incubated with anti-IRS-1 or anti-phospho-IRS-1-tyrosine (pTyr^896^) antibodies (Abcam, Cambridge, MA) (1∶1000) for 1 h at 4°C, and finally stained with FITC-secondary antibody (1∶200) for 1 h at 4°C. Samples were analyzed by flow cytometry.

#### Flow cytometry

All flow cytometric analysis was carried out using a FACSCalibur cytometer (BD Biosciences, CA). The Cell Quest software (BD Biosciences, CA) was used for data acquisition and multivariate analysis. Cells were gated in forward/side scatter dot plots and FITC or PE emission were measured in the FL1 (band pass filter 525 nm) or FL2 (band pass filter 585 nm) channels. Data on scatter parameters and histograms were acquired in log mode. Ten thousand events were evaluated for each sample and the median peak channel obtained from the histograms was used to determine levels of mIR, IRS-1, and IRS-1 tyrosine phosphorylation.

#### Determination of plasma TNF-alpha

We also determined plasma tumor necrotic factor alpha (TNF-α) levels, as these have been associated with decreased IRS-1 phosphorylation [48]. Plasma TNF-alpha levels were measured using available ELISA kits (R & D Systems) following the manufacturer's instructions. This assay detects the total amount of TNF-α in samples, which is the amount of free TNF-α plus the amount of TNF-α bound to soluble receptor. In brief, 200 µL of each sample, standards, and positive and negative controls were loaded into wells of a 96 well-plate containing antibody against human TNF-α. After addition of reagents, incubations and washes, the optical density of each well was measured using a microplate reader set to 490 nm. All samples were analyzed in triplicate and measurements repeated twice. Standard curves were run in duplicate in each assay. Mean values were obtained for each plasma sample, and means and SD were calculated for each group at each time interval. Differences were analyzed by ANOVA.

### Statistical analyses

The study was conducted to assess the association between soluble and cell-associated insulin receptor dysfunction and presence or severity of HAND. To achieve this, we compared soluble insulin receptor levels (MFI) (sIR-α and sIR-αβ) in plasma and CSF between four groups: control and HIV-seropositive women stratified by cognitive impairment into normal cognition, asymptomatic impairment, and symptomatic impairment. Because the data was not normally distributed, these comparisons were made using non-parametric Kruskal-Wallis test. Using a similar statistical technique, we compared membrane insulin receptor (mIR), insulin receptor substrate 1 (IRS-1), and IRS-1 tyrosine phosphorylation levels in CSF white cell pellets of HIV-seropositive women stratified by HAND.

The control group consisted of 5 plasma and 5 CSF samples as described earlier. The use of 5 control samples was adequate to estimate the median and interquartile range.

All statistical analyses were performed with SAS version 8.02 (SAS Institute, Cary NC) and Intercooled Stata version 8 (Stata Corp, College Station, TX).

## Results

### Soluble insulin receptor (sIR) full- length (intact, αβ subunit) and sIR ectodomain (α subunit) levels in plasma and CSF of HIV-seropositive women

**Figure 1:**
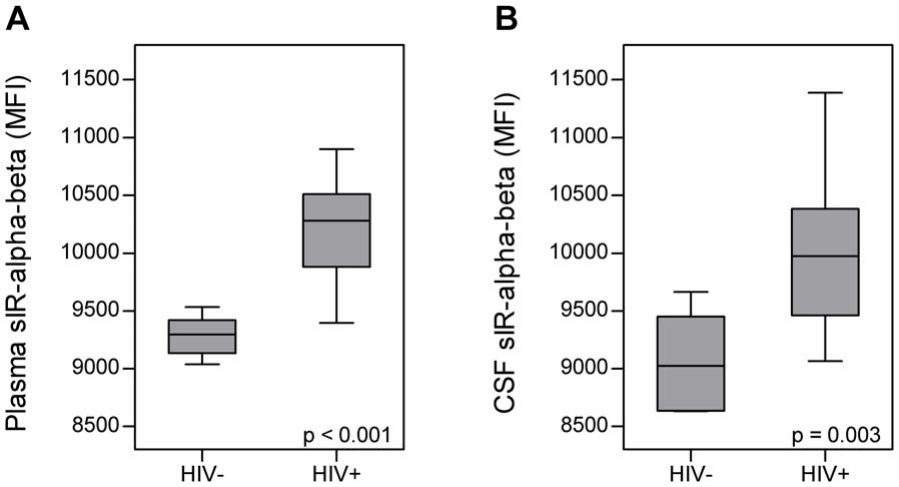
Soluble insulin receptor full-length (sIRαβ) and HIV. Soluble insulin receptor (sIR) intact or full-length (αβ) was measured in plasma (A) and CSF (B) of HIV-seropositive women (HIV+) (n = 34) and controls (HIV−) (n = 10, 5 with plasma and different 5 for CSF). The sIR subunits were determined using an ELISA [47] Significantly higher levels of full-length sIR was observed in HIV-seropositive women in plasma and CSF when compared with controls (p<0.001 and p = 0.003 respectively). (MFI = Median Fluorescence Intensity).

**Figure 2:**
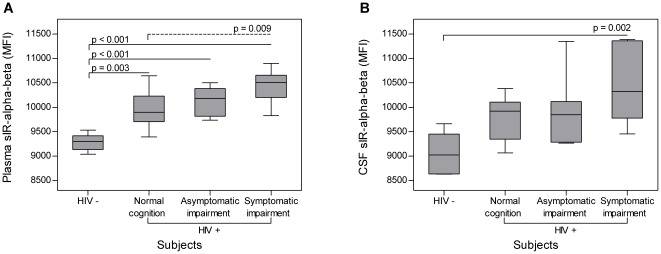
Soluble insulin receptor full-length (sIRαβ) stratified by HAND. Soluble insulin receptor (sIR) intact or full-length (αβ) subunit was measured by ELISA [47] in plasma (A) and CSF (B) of HIV-seropositive women (HIV+) (n = 34) stratified by HAND into normal cognition (n = 11), asymptomatic impairment (n = 8), and symptomatic impairment (n = 15); and 10 HIV-negative controls (HIV−) (5 plasma were different women from 5 CSF). In plasma (A), levels of full-length sIR were significantly increased from controls in all HIV-seropositive women and it correlated with the severity of HAND (normal cognition [p = 0.003], asymptomatic impairment [p<0.001], and symptomatic impairment [p<0.001]). Also, women with symptomatic impairment had significant higher levels of full-length sIR when compared to those with normal cognition (p = 0.009). A similar trend was observed in CSF samples (B), although the only significant increased was observed in the women with symptomatic impairment when compared to controls. (MFI = Median Fluorescence Intensity).

Full-length sIR levels in the plasma and CSF of HIV-seropositive women were significantly increased relative to controls (p<0.001 and p = 0.003, respectively, Figure 1). When HIV-seropositive women were stratified by HAND, higher levels of plasma full-length sIR (intact, αβ) were associated with the presence (p<0.001) and severity of HAND (p<0.001) (Figure 2A, Table 3). Similar results were observed for CSF sIR-αβ levels (p = 0.005 with the presence and p = 0.002 with the severity of HAND) (Figure 2B). No significant differences were observed in plasma and CSF sIR ectodomain (α) levels.

**Table 3:** Soluble Insulin Receptor Levels (MFI) and HIV-seropositive women stratified by HAND.

sIR levels (MFI)^a^	Control (n = 10) (5/group)	Normal cognition (n = 11)	Asymptomatic impairment (n = 8)	Symptomatic impairment (n = 15)	p value^b^
Plasma sIRαβ	9296 (9230, 9307)	9900 (9707, 9989)	10181 (9844.5, 10373)	10510 (10330, 10649)	<0.001^*^
Plasma sIRα	10291 (9880, 10301)	9746 (9154, 10189)	9920.5 (9435, 10391)	10195 (9477, 10657)	0.599
CSF sIRαβ	9024 (8637, 9237)	9921 (9353, 9977)	9846 (9286, 10115)	10327 (9780, 11361)	0.005^*^
CSF sIRα	9473 (9291, 10165)	9758 (9267, 10058)	9746 (9230, 9987)	9786 (9423, 9961)	0.973

a MFI  =  Median Fluorescence Intensity; median (interquartile range [25^th^ and 75^th^ percentile]),

b significant p value<0.05^*^.

### Membrane-bound insulin receptor (mIR), insulin receptor substrate-1 (IRS-1), and IRS-1 tyrosine phosphorylation levels in white blood cells (WBC) from the CSF of HIV-seropositive women

**Figure 3:**
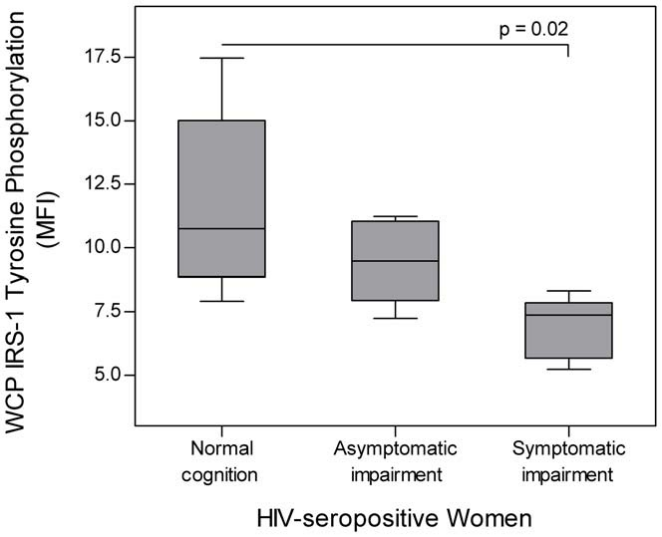
Insulin Receptor Substrate 1 (IRS-1) tyrosine phosphorylation stratified by HAND. Insulin receptor substrate 1 (IRS-1) tyrosine phosphorylation was determined in CSF cell pellets of 23 HIV-seropositive women (HIV+) stratified by HAND (7 with normal cognition, 7 with asymptomatic impairment, and 9 with symptomatic impairment) using flow cytometry. A significant decrease in IRS-1 tyrosine phosphorylation was observed between HIV-seropositive women with normal cognition and symptomatic impairment (p = 0.02). (MFI = Median Fluorescence Intensity).

No significant differences were observed in the mIR and IRS-1 levels of HIV-seropositive women stratified by HAND (p = 0.834 and 0.868 respectively). A significant decrease in IRS-1 tyrosine phosphorylation was observed in HIV-seropositive women with symptomatic impairment versus those with normal cognition (p = 0.02, Table 4 and Figure 3).

**Table 4:** Membrane insulin receptor (mIR), insulin receptor substrate 1 (IRS-1), and IRS-1 tyrosine phosphorylation levels in the CSF white cell pellet (CSF WCP) stratified by HAND.

CSF WCP^a^	Normal cognition (n = 11)	Asymptomatic impairment (n = 8)	Symptomatic impairment (n = 15)	p value^b^
mIR (MFI)^c^	7.42 (5.71, 9.93)	8.06 (7.64, 9.14)	8.10 (6.32, 10.84)	0.834
IRS-1	15.82 (14.86, 25.48)	18.11 (17.00, 20.54)	17.78 (16.55, 20.44)	0.868
IRS-1 tyrosine phosphorylation	10.75 (9.82, 12.55)	9.47 (8.66, 10.84)	7.37 (6.10, 7.37)	0.027^*^

a For the mIR (MFI) and IRS-1 we analyzed 11 women with normal cognition, 8 with asymptomatic impairment, and 15 with symptomatic impairment. For IRS-1 tyrosine phosphorylation 5 women per group were analyzed;

b significant p value<0.05^*^;

c MFI = Median Fluorescence Intensity; median (interquartile range [25^th^ and 75^th^ percentile]).

### Plasma tumor necrotic factor alpha (TNFα) determination

No association was observed between levels of TNFα and cognitive performance (p = 0.325, data mot shown) and no correlation was observed between levels of TNFα and levels of IRS-1 tyrosine phosphorylation (p = 0.43, data not shown).

## Discussion

While the prevalence of severe HAND has decreased in the CART era, that of the milder forms has increased, and cognitive deterioration and neuropathological changes progress despite viral suppression and curtailment of immunosuppression by CART [5], [8], [49], [50], [51], [52]. These changes could result from inadequate penetration of CART and residual infection in the brain, chronic CNS inflammatory and oxidative stress responses, and/or neurotoxic effects of long-term CART treatment [52], [53], [54]. Even with advances in HIV treatment and increased patient survival, the presence of HAND is associated with increased morbidity and mortality [1], [55]. Hence, there is an imperative need to understand the pathophysiology of HAND and identify new biomarkers for HAND [9], [10], [32], [50].

The Soluble Insulin Receptor Study Group designed a method to determine plasma levels of two forms of the soluble insulin receptor: ectodomain (α) and full-length or intact protein (αβ). They found that patients with diabetes type 1 had significantly increased levels of the ectodomain, but not the full-length sIR [47]. They proposed the use of the sIR ectodomain as biomarker for hyperglycemic states. In contrast, our data suggest that levels of full-length sIR in plasma could be associated with asymptomatic glucose derangements in HIV-seropositive women using CART. In diabetes type 1, sIR ectodomain (α) is likely to occur due to the cleavage of the ectodomain of the receptor from cell surfaces [47]. In contrast, the appearance of intact or full-length sIR in plasma probably occurs by a different mechanism, such as release from damages tissues or mechanisms akin to the generation of soluble cytokine receptors [56], [57].

Several cohort studies observed an association between insulin resistance, diabetes, and metabolic syndrome in HIV-seropositive individuals whose CART regimen included protease inhibitors [14], [15], [58], [59], [60]. However those cohorts were composed mostly of men, and studies of cohorts of women have produced conflicting findings [61], [62], [63], [64]. In this study, we observed no associations between the use of CART, use of protease inhibitors, or the CPE with either IR dysfunction or HAND. Hence, there is a need to study this issue further in women, and identify factors that could contribute to the development of insulin resistance in HIV-seropositive women, such as IR dysfunction.

We observed a significant positive association between full-length sIR levels and the presence and severity of HAND in a cohort of HIV-seropositive women without history of abnormal glucose metabolism. These findings may suggest that asymptomatic glucose abnormalities as determined by a significant decrease in IRS-1 tyrosine phosphorylation may impact cognitive function in HIV-seropositive women [65], [66].

The brain is no longer considered an insulin-insensitive organ. It has been shown that insulin crosses the blood brain barrier (BBB) and that insulin receptors are widely distributed in the brain. Brain IRs are found on both astrocytes and neurons [22], [23], [24], and in the human brain, the highest density of IR is found in the hippocampus and cerebral cortex [22]. Insulin receptor and the type 1 insulin-like growth factor receptor have been shown to have essential roles in energy homeostasis, neuronal growth and survival, and synaptic plasticity (Reviewed in [20], [22], [23], [24]). A difference has been observed between brain and peripheral IRs, with the brain IR having smaller alpha (α) subunit [22], [24]. However, once the brain IR is activated by insulin the signaling effects are the same as in the periphery. Hence, its activation will mediate insulin-induced glucose transport into neurons and activate the signal-transduction pathway for energy production and protein synthesis [22].

We observed a significant increase in levels of CSF sIR in women with HAND. sIR in the CSF which may be generated by several mechanisms, including secretion from astrocytes, neurons, or PBMCs that cross the BBB, brain tissue injury, or plasma sIR that crosses the BBB. In our study CSF sIR levels were similar to plasma sIR levels, suggesting a passive transfer from the blood. However, we can not exclude the possibility of brain IR contribution.

This retrospective study has some limitations. First, we assessed sIR but not blood sugar metabolism (e.g., by the oral glucose tolerance test, glycosylated hemoglobin assay, or homeostasis model assessment). However, the observation of decreased IRS-1 tyrosine phosphorylation is consistent with insulin resistance [65], [66]. Second, when determining HAND using a reference control group for normative purposes, we observed a significant difference in age between HIV-seropositive women and controls (p = 0.001), with the reference control group being younger. That finding raised the possibility that increased age might contribute to the increased level of cognitive impairment seen in the HIV-seropositive group. However, when the HIV-seropositive group was stratified by the presence of HAND, there was no significant association of age with degree of cognitive impairment. If the observed cognitive impairment in the HIV-seropositive women was due to the younger reference group (controls for NP z-scores), this effect should be similar across HAND groups and therefore should not alter the sIR study results. It should be noted that the sIR study control group was relatively small (consisting of 5 plasma and 5 CSF samples). Nonetheless, the use of 5 control samples was adequate to estimate the median and interquartile range.

This study provides evidence that IR secretion is increased in HIV-seropositive women, and increased IR secretion is associated with cognitive impairment in these women. Our findings suggest that full-length sIR in plasma could be associated with asymptomatic glucose derangements in HIV-seropositive women using CART and is a contributing factor in the development and progression of HAND. Since early intervention may help prevent the progression from mild to more severe forms of HAND, it is vital to identify an easily measurable and reliable biomarker for HAND. Full-length sIR may serve as a blood biomarker for the presence and severity of HAND.
